# Outbreak of West Nile Virus Infection in Greece, 2010

**DOI:** 10.3201/eid1710.110525

**Published:** 2011-10

**Authors:** Kostas Danis, Anna Papa, George Theocharopoulos, Georgios Dougas, Maria Athanasiou, Marios Detsis, Agoritsa Baka, Theodoros Lytras, Kassiani Mellou, Stefanos Bonovas, Takis Panagiotopoulos

**Affiliations:** Hellenic Centre for Disease Control and Prevention, Athens, Greece (K. Danis, G. Theocharopoulos, G. Dougas, M. Athanasiou, M. Detsis, A. Baka, T. Lytras, K. Mellou, S. Bonovas, T. Panagiotopoulos);; Aristotle University of Thessaloniki, Thessaloniki, Greece (A. Papa);; National School of Public Health, Athens (T. Panagiotopoulos)

**Keywords:** West Nile virus, viruses, neuroinvasive disease, encephalitis, meningitis, paralysis, outbreak, Greece, dispatch

## Abstract

During 2010, an outbreak of West Nile virus infection occurred in Greece. A total of 197 patients with neuroinvasive disease were reported, of whom 33 (17%) died. Advanced age and a history of heart disease were independently associated with death, emphasizing the need for prevention of this infection in persons with these risk factors.

An outbreak of West Nile virus (WNV) infection occurred in Central Macedonia in northern Greece in the summer of 2010. The first cases were diagnosed and reported to the Hellenic Centre for Disease Control and Prevention (HCDCP) on August 5, 2010 (*1*). WNV lineage 2 sequences were later obtained from 3 pools of *Culex pipiens* mosquitoes trapped at 2 sites where cases of West Nile neuroinvasive disease (WNND) had occurred (*2*).

Human cases of WNV disease had not been previously reported in Greece. Serosurveys in the early 1960s, 1980s, and 2007 identified WNV antibodies in ≈1% of the population, suggesting that WNV, or a related flavivirus, was circulating in Greece (*3**–**5*). In contrast, during 2005–2007, a total of 9,590 blood donors were tested by WNV nucleic acid amplification assay and results were negative (*6*).

## The Study

After the outbreak alert was issued in early August 2010, physicians in Greece were asked to report all cases of WNV infection to HCDCP, according to the current European Union case definition (*1*). Only deaths that occurred during hospitalization were attributed to WNV infection. Statistical methods are described in the Technical Appendix.

Serum and cerebrospinal fluid specimens were tested for immunoglobulin (Ig) M and IgG against WNV by using an ELISA (WNV IgM capture DxSelect and WNV IgG DxSelect; Focus Diagnostics Inc., Cypress, CA, USA). Titers >640 were obtained by microneutralization assay against WNV in 14/14 patients who were positive for WNV. Although tick-borne encephalitis virus (TBEV) and dengue virus (DENV) are not prevalent in Greece, specimens were also tested for TBEV and DENV by ELISA (TBE/FSME IgM and TBE/FSME IgG; IBL International Gmbh, Hamburg, Germany) and Dengue Virus IgM Capture Dx Select and IgG Dx Select (Focus Diagnostics Inc.). All specimens were negative for TBEV, and cross-reactivity was seen with DENV, mainly for IgM (*7*).

Overall, 262 patients with WNV infection were reported to HCDCP. Of these patients, 197 (75%) had neuroinvasive disease (encephalitis, meningitis, or acute flaccid paralysis), and 65 (25%) had WNV fever. This study focused on patients with WNND, who were identified and reported more consistently because of disease severity.

**Figure 1:**
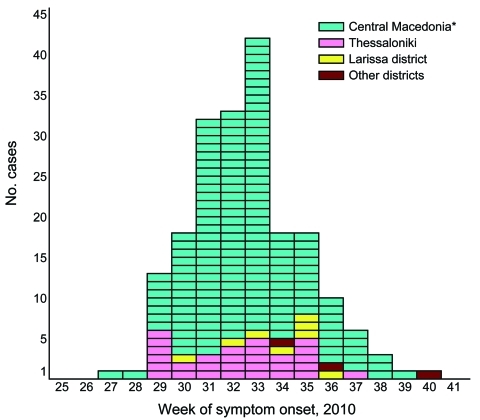
Reported cases (n = 197) of West Nile neuroinvasive disease, by week of symptom onset, Greece, July 6–October 5, 2010. *Excluding Thessaloniki.

**Figure 2:**
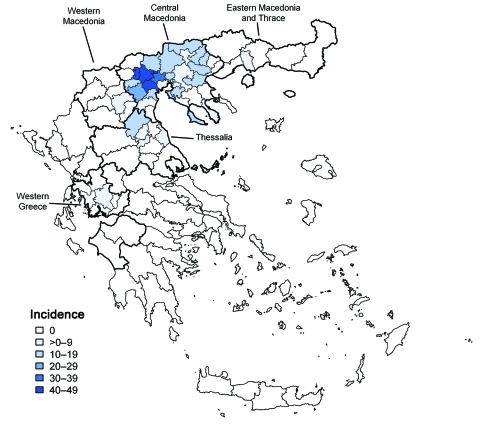
Incidence per 100,000 population of 197 reported cases of West Nile neuroinvasive disease, by township of residence, Greece, July–October 2010. Districts with >1 reported neuroinvasive cases were divided into townships. Dark black lines indicate borders of Central Macedonia (north) and Thessalia (south).

Patient disease onset occurred within a 14-week interval during July 6–October 5, and the outbreak peaked in mid August (Figure 1). Most (94%) patients with WNND were reported from the 7 districts of Central Macedonia (Figure 2), and the epicenter of the outbreak was in Pella and Imathia Districts.

Characteristics of patients with WNND are shown in Table 1. Median age of patients with neuroinvasive disease was 72 years (range 12–88 years). The attack rate for WNND increased significantly (p = 0.006) with age (Table 1). The incidence of WNND in older persons (>80 years of age) was ≈50× higher than that among the youngest age group (<20 years of age). Persons living in rural areas were 2× as likely to show development of WNND than persons living in urban areas (Table 1).

**Table 1:** Characteristics of 197 patients with West Nile neuroinvasive disease. Greece, July–October 2010*

Characteristic	No. patients	Incidence per 100,000 population	Risk ratio (95% CI)
Age group, y			
<20	4	0.18	Reference
20–29	3	0.20	1.08 (0.24–4.84)
30–39	6	0.34	1.87 (0.53–6.63)
40–49	9	0.55	3.03 (0.93–9.82)
50–59	18	1.27	6.93 (2.35–20.49)
60–69	29	2.44	13.31 (4.68–37.84)
70–79	85	8.01	43.74 (16.05–119.2)
>80	43	9.63	52.62 (18.89–146.6)
Sex			
F	88	1.59	Reference
M	109	1.97	1.26 (0.95–1.67)
Place of residence			
Urban	110	1.38	Reference
Rural	87	2.92	2.12 (1.60–2.80)
Districts in Central Macedonia			
Chalkidiki	4	3.99	0.76 (0.28– 2.09)
Thessaloniki	60	5.27	Reference
Pieria	9	7.02	1.33 (0.66–2.69)
Serres	21	11.15	2.12 (1.29–3.48)
Kilkis	12	13.92	2.64 (1.42–4.91)
Imathia	39	27.06	5.14 (3.43–7.69)
Pella	41	28.26	5.37 (3.61–7.98)
Total	186	15.00	NA
Other districts (region)			
Etoloakarnania (western Greece)	1	0.46	0.09 (0.01–0.63)
Kozani (Western Macedonia)	1	0.65	0.12 (0.02–0.89)
Kavala (Eastern Macedonia)	1	0.71	0.14 (0.02–0.98)
Larissa (Thessalia)	8	2.80	0.53 (0.25–1.11)
Total in Greece	197	1.76	NA

*Incidence rates were calculated by using 2008 mid-year population estimates of the Hellenic Statistical Authority as the denominator. CI, confidence interval; NA, not applicable.

Encephalitis/meningoencephalitis (168 patients, 85%) was the most prominent clinical syndrome among patients with WNND, followed by meningitis (23, 12%). In addition, 10 (5%) patients with acute flaccid paralysis were reported, 6 (3%) of whom did not have meningitis or encephalitis.

A large proportion (74%) of patients with WNND had >1 underlying chronic medical condition; the most common were hypertension (39%), heart disease (24%), diabetes mellitus (24%), and immunosuppression (10%). Patients with WNND were 2× more likely (odds ratio 2.16, 95% confidence interval 1.15–4.04) than patients without WNND to have underlying conditions.

Thirty-five patients died during hospitalization (33 had WNND), indicating an overall case-fatality rate of 17% among persons with WNND. Median age of WNND patients who died was 78 years (range 49–87 years). The case-fatality rate increased substantially (p<0.001) with age (Table 2). Median interval from WNV disease onset to death was 13 days (range 3–90 days). In 15 (45%) patients with WNND who died, the interval between disease onset and death exceeded 2 weeks.

**Table 2:** Predictive factors of death for 197 patients with West Nile neuroinvasive disease analyzed by univariate and multivariate analysis, Greece, July–October 2010*

Characteristic	No. deaths, n = 33†	Case-fatality rate, %	Crude risk ratio (95% CI)	Adjusted risk ratio‡ (95% CI)
Age group, y				
40–59	1§	2.50	Reference	Reference
60–69	2	6.90	2.76 (0.26–28.99)	2.72 (0.26–28.40)
70–79	15	17.65	7.06 (0.97–51.59)	6.13 (0.83–45.17)
>80	15	34.88	13.95 (1.93–100.9)	11.41 (1.56–83.52)
Sex				
F	10	11.36	Reference	NA
M	23	21.10	1.86 (0.93–3.69)	NA
Underlying diseases				
None	2	3.92	Reference	NA
>1	31	21.23	5.41 (1.34–21.82)	NA
Hypertension				
No	19	15.70	Reference	NA
Yes	14	18.42	1.17 (0.63–2.20)	NA
Heart disease				
No	18	12.00	Reference	Reference
Yes	15	32.61	2.72 (1.49–4.95)	2.03 (1.14–3.64)
Diabetes				
No	23	15.44	Reference	NA
Yes	10	20.83	1.35 (0.69–2.63)	NA
Immunosuppression				
No	31	17.42	Reference	NA
Yes	2	10.53	0.60 (0.16–2.33)	NA
Cancer				
No	29	16.11	Reference	NA
Yes	4	23.53	1.46 (0.58–3.66)	NA
Stroke				
No	28	15.30	Reference	NA
Yes	5	35.71	2.33 (1.07–5.10)	NA
Renal failure				
No	31	16.40	Reference	NA
Yes	2	25.00	1.52 (0.44–5.28)	NA

*CI, confidence interval; NA, not applicable. †Two additional patients with nonneuroinvasive disease died, and those deaths were not included in this analysis. ‡In logistic regression analysis, initial models included all variables for which the p value was <0.05 or the odds ratio was >1.1 or <0.90. Therefore, all variables were included in the initial models. Variables were removed 1 at a time depending on results of statistical testing (p<0.05), by using the likelihood-ratio test. All variables that remained significant in the final logistic regression model were included in the binomial regression model for the estimation of adjusted risk ratios. § Belonged to the 40–49-year age group.

WNND patients with >1 underlying disease were 5× more likely to have died than patients without underlying conditions. Those patients who had heart disease or a stroke were ≈2.5× more likely to have died than patients without these conditions. However, only older age and heart disease were independent predictors of death in the final binomial regression model (Table 2). Supplementary results are shown in the Technical Appendix.

## Conclusions

Human cases of WNV infection were detected in several European and Mediterranean countries in 2010, indicating an increased intensity of viral circulation (*8*). Clinical cases of WNV infection in humans or animals had not been previously reported in Greece. The present outbreak was the largest in Europe since 1996, when a large outbreak was observed in Romania (*9*). The outbreak was located in Central Macedonia, which contains 90% of the rice paddies and 70% of the wetland areas in Greece and provides a favorable environment for reproduction of mosquito vectors (*8*). The region also hosts one of the largest populations of migratory birds in Greece. Meteorologic data for the area indicate that 2010 was warmer than previous years and unusually wet (*8*).

The overall case-fatality rate among patients with WNND (17%) was higher in Greece than that in other countries (*9**–**11*). The reasons for this finding are not clear. Many factors may have played a role in differences in the fatality rate. These factors include diagnosis and surveillance bias for more severe cases, virus strain, host susceptibility, age structure of the population, and underlying conditions.

Recent studies on WNV lineage 2 suggested that this virus may be underestimated as a cause of neuroinvasive disease (*2**,**12*). WNV linage 2 isolated from *Cx. pipiens* mosquitoes in the affected areas during this outbreak had a nucleotide genetic similarity of 99.6% with the goshawk Hungary 2004 strain (*12*). However, few severe cases of WNV infection were reported in Hungary. Experimental studies would verify whether the amino acid substitution H249P detected in the Greek strain, which is a suspected virulence marker in lineage 1 strains, is associated with increased virulence (*12*).

Advanced age and heart disease were found to independently predict the risk for WNND-related death. The association between age and severe disease has been reported (*9**–**11*). The contributing factor of age may relate to a decrease in the integrity of the blood–brain barrier and facilitate access of WNV to the central nervous system (*13*). Heart disease, particularly cardiac arrhythmias, have also been recognized as frequent contributors to death caused by WNV encephalitis (*13**–**15*). Physiologic stress of WNV infection may precipitate or exacerbate underlying medical conditions resulting in death (*14*).

These findings emphasize the need for primary prevention of WNV infection in patients with these predisposing conditions and close monitoring for cardiac complications in elderly patients hospitalized with WNV disease. Vector mosquito control programs, including source reduction and larviciding of *Culex* spp. mosquitoes and ongoing public health education and WNV surveillance in disease-endemic and newly affected areas, remain the cornerstones of WNV disease prevention and control.

## Supplementary Material

Technical Appendix Incidence rates were calculated by using the 2008 mid-year population estimates of the Hellenic Statistical Authority as the denominator.
