# Supplementary material for: Outbreak of West Nile Virus Infection in Greece, 2010
Source: Emerg Infect Dis. 2011 Oct;17(10):1868–72. doi: 10.3201/eid1710.110525 (PMC3310677; doi:10.3201/eid1710.110525)
Supplement: Technical Appendix — Incidence rates were calculated by using the 2008 mid-year population estimates of the Hellenic Statistical Authority as the denominator. [file 11-0525-Techapp_2p.pdf]

Article DOI: http://dx.doi.org/10.3201/eid1710.110525

Outbreak of West Nile Virus Infection in Greece, 2010

Technical Appendix

Statistical Methods

Incidence rates were calculated by using the 2008 mid-year population estimates of the Hellenic Statistical Authority as the denominator. Definitions of this authority were also used for urban and rural areas; townships with populations >2,000 persons were classified as urban. Comparison of categorical variables was assessed by using the χ^2 test. Risk ratios (RRs) were calculated to compare incidence rates and to identify predictors of fatal outcome. Multiple logistic regression models were constructed to identify independent factors associated with deaths caused by West Nile neuroinvasive disease (WNND). In logistic regression analysis, initial models included all variables for which $p<0.05$ or the odds ratio was >1.10 or <0.90. To simplify the models, variables were removed 1 at a time depending on statistical testing ($p<0.05$) by using the likelihood-ratio test. Adjusted RRs were estimated from binomial regression that included all variables that remained significant in the final logistic regression model. Hosmer-Lemeshow goodness-of-fit statistic was used to evaluate the fit of the final model. The analysis was conducted by using STATA version 10 software (StataCorp LP, College Station, TX, USA). Incidence data were mapped by township and district (prefecture) by using GNU R software (www.gnu.org/s/r/).

Geographic Spread of the Outbreak

The first cases occurred in Central Macedonia in northern Greece. The outbreak evolved and peaked almost simultaneously in rural and urban parts of the region, including the city of Thessaloniki, the second largest city in Greece. Face-to-face interviews showed that that 17 of 29 patients with WNND among Thessaloniki residents had no travel history to another affected area, suggesting that transmission occurred in the city. Three weeks after the beginning of the outbreak, it spread southward to the adjacent Larissa district (Thessalia region). After the peak of

the epidemic, sporadic cases occurred in 3 other regions, but there was no evident epidemiologic link with the other locations.

Hospitalization and Intensive Care Unit Admission

All but 1 of the reported patients with WNND were hospitalized, and 22 (12%) were admitted to an intensive care unit (ICU). The median length of stay in hospital for patients with WNND was 12 days (range 3–75 days) and was significantly ($p = 0.034$) higher than that for patients with non-neuroinvasive disease (9 days, range 3–39 days). Of all reported patients with non-neuroinvasive disease, 54 (83%) were hospitalized, 2 (3%) in an ICU. All 33 patients who died of WNND were hospitalized, of whom 13 (39%) were in an ICU.

Neurologic Manifestations and Age

The median age (74 years, range 19–88 years) of patients with encephalitis or meningoencephalitis was significantly higher ($p<0.001$) than that of patients with only meningitis (64 years, range 12–80 years). Thus, younger patients were more likely to have only meningitis, with the risk for meningitis decreasing by ≈4% (RR 0.96, 95% confidence interval 0.95–0.98) for every 10-year increase in age. The median age of patients with acute flaccid paralysis was 74 years (range 63–81 years).

Symptoms

The most common symptom of patients with neuroinvasive disease was fever (187, 95%), followed by headache (104, 53%), weakness (57, 29%), and nausea/vomiting or diarrhea (35, 18%). The distribution of reported signs and symptoms did not differ between patients with neuroinvasive disease and those with non-neuroinvasive disease.
